# A one-year genomic investigation of *Escherichia coli* epidemiology and nosocomial spread at a large US healthcare network

**DOI:** 10.1186/s13073-022-01150-7

**Published:** 2022-12-30

**Authors:** Emma G. Mills, Melissa J. Martin, Ting L. Luo, Ana C. Ong, Rosslyn Maybank, Brendan W. Corey, Casey Harless, Lan N. Preston, Joshua A. Rosado-Mendez, Scott B. Preston, Yoon I. Kwak, Michael G. Backlund, Jason W. Bennett, Patrick T. Mc Gann, Francois Lebreton

**Affiliations:** 1 Multidrug-Resistant Organism Repository and Surveillance Network, Walter Reed Army Institute of Research, Silver Spring, MD USA; 2 Department of Pathology, Walter Reed National Military Medical Center, Bethesda, MD USA

**Keywords:** *Escherichia coli*, Genomic epidemiology, ST-131, Antibiotic resistance, Nosocomial

## Abstract

**Background:**

Extra-intestinal pathogenic *Escherichia coli* (ExPEC) are a leading cause of bloodstream and urinary tract infections worldwide. Over the last two decades, increased rates of antibiotic resistance in *E. coli* have been reported, further complicating treatment. Worryingly, specific lineages expressing extended-spectrum *β*-lactamases (ESBLs) and fluoroquinolone resistance have proliferated and are now considered a serious threat. Obtaining contemporary information on the epidemiology and prevalence of these circulating lineages is critical for containing their spread globally and within the clinic.

**Methods:**

Whole-genome sequencing (WGS), phylogenetic analysis, and antibiotic susceptibility testing were performed for a complete set of 2075 *E. coli* clinical isolates collected from 1776 patients at a large tertiary healthcare network in the USA between October 2019 and September 2020.

**Results:**

The isolates represented two main phylogenetic groups, B2 and D, with six lineages accounting for 53% of strains: ST-69, ST-73, ST-95, ST-131, ST-127, and ST-1193. Twenty-seven percent of the primary isolates were multidrug resistant (MDR) and 5% carried an ESBL gene. Importantly, 74% of the ESBL*-E.coli* were co-resistant to fluoroquinolones and mostly belonged to pandemic ST-131 and emerging ST-1193. SNP-based detection of possible outbreaks identified 95 potential transmission clusters totaling 258 isolates (12% of the whole population) from ≥ 2 patients. While the proportion of MDR isolates was enriched in the set of putative transmission isolates compared to sporadic infections (35 vs 27%, *p* = 0.007), a large fraction (61%) of the predicted outbreaks (including the largest cluster grouping isolates from 12 patients) were caused by the transmission of non-MDR clones.

**Conclusion:**

By coupling in-depth genomic characterization with a complete sampling of clinical isolates for a full year, this study provides a rare and contemporary survey on the epidemiology and spread of *E. coli* in a large US healthcare network. While surveillance and infection control efforts often focus on ESBL and MDR lineages, our findings reveal that non-MDR isolates represent a large burden of infections, including those of predicted nosocomial origins. This increased awareness is key for implementing effective WGS-based surveillance as a routine technology for infection control.

**Supplementary Information:**

The online version contains supplementary material available at 10.1186/s13073-022-01150-7.

## Background

Extra-intestinal pathogenic *Escherichia coli* (ExPEC) are a leading cause of healthcare-associated urinary tract and bloodstream infections [1, 2]. Diseases caused by multidrug-resistant (MDR) strains are associated with poor patient outcomes, including high morbidity and mortality, and higher healthcare costs [3–5]. In recent years, resistance to commonly prescribed antibiotics has increased in *E. coli* infections in the USA [e.g., 1.2 to 25% prevalence of fluoroquinolone resistance in the past 15 years [6, 7]] and internationally [1, 3, 8, 9]. Importantly, resistance to 3rd- and 4th-generation cephalosporins, due to the acquisition and horizontal spread of extended-spectrum *β*-lactamase (ESBL) genes, has increased in both healthcare and community settings [10]. This alarming rise prompted the US Centers for Disease Control and Prevention to identify the ESBL-producing *E. coli* as a serious threat and urging increased surveillance efforts [11].

Previous molecular studies have separated *E. coli* into phylogenetic groups, including A, B1, B2, C, D, E, and F, with ExPEC (and consequently the specialized uropathogenic [UPEC] pathotype) largely belonging to phylogroups B2 and D [12, 13]. Multilocus sequence typing (MLST) provides further characterization of *E. coli* lineages and has led to the identification of specific, globally distributed sequence types (STs). For example, the ST-131 ExPEC lineage is widely distributed and associated with the emergence of fluoroquinolone resistance and frequent carriage of plasmid-bound ESBL genes [12, 14–17]. Besides resistances, recent studies suggest that the acquisition of virulence-associated genes also plays an integral role in the success and global emergence of ST-131 and other ExPEC lineages. These include a plethora of both structural (e.g., fimbriae, pili, curli, flagella) and secreted (e.g., toxins, iron-acquisition systems) virulence factors often enriched in non-MDR, UPEC lineages (e.g., ST-73, 95, and 127) [18–20].

The recent positioning of whole-genome sequencing (WGS) as a near-routine technology is creating a revolution in infection control and allows for targeted interventions to reduce the burden of healthcare-associated infections (HAIs). Such effort requires an understanding of the frequency of nosocomial transmission caused not only by MDR epidemic clones, but also by the more ubiquitous non-MDR lineages. While the latter are responsible for most *E. coli* infections, very few genome-based studies have examined their role in nosocomial transmission. Instead, most investigations have been performed on small cohorts, often limited to ESBL-producing isolates, which likely underrepresents the extent of *E. coli* nosocomial transmission events [21].

Here, we retrospectively genome-sequenced and analyzed a complete set of 2075 *E. coli* clinical isolates collected from 1776 patients over a 12-month period from a large military healthcare network in the Northeast United States. Genome-based detection of possible outbreak clusters revealed extensive roles for non-MDR lineages in suspected nosocomial transmissions, while in-depth phylogenetic, genotypic, and phenotypic characterization revealed a detailed picture of the epidemiology, population structure, and prevalence of resistances in *E. coli* in this region.

## Methods

### Isolation and phenotypic characterization of *E. coli* collection

A total of 2075 *E. coli* isolates (including serial isolates from the same patient) cultured from all clinical specimens of 1776 patients receiving care in the National Capitol Medical Region healthcare network between October 2019 and September 2020 were collected. Of note, no stool isolates were collected as these samples are not routinely sent for culture in the microbiology lab of this hospital and are instead analyzed by molecular and/or antigen diagnostic procedures. Antibiotic susceptibility testing (AST) was performed in a College of American Pathologists (CAP)-certified laboratory using the BD Phoenix (panel NMIC/ID304; BD Diagnostics), which encompasses 18 antibiotics from 11 different antibiotic classes. Where necessary, MICs were determined in triplicate using broth microdilution using Clinical and Laboratory Standards Institute (CLSI) guidelines [22]. Breakpoints were interpreted using CLSI guidelines (2018), with cefazolin MICs interpreted using breakpoints for complicated UTI/systemic infection [22]. Isolates with breakpoints interpreted as I or R were designated non-susceptible. To accurately calculate the prevalence of resistances in the population, a subset of 1828 primary isolates (first isolate of each ST per patient) was specifically used (Table 1).

**Table 1:** Prevalence of MDR and key resistances in *E. coli* isolates

	Total (***n***)	MDR (%)	Non-MDR (%)	ESBL^b^ (%)	FLQ^c^ (%)
**All**	2075	29	71	6	17
Cluster	258	36	64	6	23
Non-cluster	1817	28	72	6	16
**Deduplicated**^a^	**1828**	**27**	**73**	**5**	**15**
Cluster	228	35	65	6	21
Non-cluster	1600	26	74	5	14
**B2**	**1290**	**26**	**74**	**4**	**16**
Cluster	186	33	67	5	22
Non-cluster	1104	25	75	4	15
**ST-131**	215	64	36	16	52
Cluster	36	72	28	22	56
Non-cluster	179	63	37	13	49
**ST-73**	204	21	79	1	0
Cluster	41	29	71	5	0
Non-cluster	163	20	80	1	1
**ST-95**	196	7	93	0	0
Cluster	21	24	76	0	0
Non-cluster	175	5	95	0	0
**ST-127**	125	13	87	0	0
Cluster	21	24	76	0	0
Non-cluster	104	11	89	0	0
**ST-1193**	89	55	45	7	100
Cluster	17	53	47	0	100
Non-cluster	72	56	44	9	100
**D**	**243**	**40**	**60**	**8**	**11**
Cluster	42	48	52	7	12
Non-cluster	201	39	61	8	10
**ST-69**	128	43	57	4	6
Cluster	21	47	53	0	4
Non-cluster	107	42	58	5	6

^a^Deduplicated = primary isolate of each ST per patient

^b^Presence of extended-spectrum β-lactamases

^c^Phenotypic non-susceptibility to ciprofloxacin and levofloxacin

### Whole-genome sequencing

DNA extraction and WGS were performed as previously described [23]. In brief, genomes were generated for all 2075 isolates using an Illumina MiSeq platform with a 2×300 nt paired-end protocol or a NextSeq-500 platform with a 2×150 nt paired-end protocol. Libraries were prepared using the Kapa HyperPlus kit (Roche Diagnostics) and quantified using the Kapa library quantification kit Illumina/Bio-Rad iCycler (Roche Diagnostics) on a CFX96 real-time cycler (Bio-Rad). De novo assemblies were obtained using Newbler v2.7 (Roche Diagnostics). Minimum thresholds for contig size and coverage were set at 200 bp and 49.5+, respectively. Assembled sequences were annotated using Prokka v1.14.6 [24].

### Bioinformatic analysis

Species identification was determined using Kraken2 (v2.0.8-β) [25] and *E. coli* phylogenetic groups were identified using EzClermont v0.6.3 [26, 27]. In silico ST detection was identified for all isolates using the Achtman MLST scheme through software [28]. This tool uses the PubMLST website [29] developed by Keith Jolley and sited at the University of Oxford. Novel ST was assigned using the MLST sequence archive at EnteroBase [30]. Serotyping and *fimH* typing were performed using the TORMES pipeline v1.3.0 [31] with the SerotypeFinder O-typing database [32] and FimTyper [33], respectively. Antimicrobial resistance genetic determinants were annotated using AMRFinderPlus [34] and ARIBA [35]. Plasmid replicons [36] and virulence-associated genes (based on the *E. coli* virulence-associated gene databases EcVGDB and VFDB [37, 38]) were identified using ABRicate [39].

### Phylogenetic analysis

For the phylogeny of the diverse set of 123 ESBL-*E. coli*, the annotated [Prokka v1.14.6 [24]] assemblies were used as input for Roary v3.13.0 [40] and a SNP-based alignment of 2698 core genes was generated. For the phylogeny of the clonal set of 275 ST-131 *E.* coli, SNP calling was performed with Snippy v.4.4.5 [41] using error correction [Pilon v1.23 [42]] and the annotated genome of ST-131 *E. coli* EC958 (accession no. GCA_000285655.3) as a reference. For both approaches, recombination was filtered from the alignments using Gubbins v2.4.1 [43] and a maximum-likelihood tree was generated with RAxML v8.2.12 [44] using the GTR+G (50 parsimony, 50 random) model and 100 random bootstrap replicates. Trees were imported in iTOL v.5.5 [45] for visualization with metadata.

Finally, for the ST-131 phylogenetic analysis, clade designations (A, B, and C) were generally characterized by the carriage of type 1 fimbriae adhesion *fimH* alleles (*fimH*41, *fimH*22, and *fimH*30, respectively) and subclades C0, C1, and C2 based on SNP typing of genetic markers *gyrA*, *parC*, and *ybbW* genetic markers, as previously described [16, 46]. The G273A SNP *in ybbW* (subclade C2-specific allele) was identified using an individual gene alignment produced by Roary v3.13.0 [40].

### Nosocomial transmission analysis

Detection of clusters of transmission was performed in two stages. First, cgMLST allele assignment and minimum spanning tree generation were performed with SeqSphere+ [47] using the *E. coli* cgMLST scheme developed by Zhou et al. [30]. The distance matrix from SeqSphere+ consisted of the pairwise allelic differences between all 2075 *E. coli* isolates*.* Using a threshold of ≤10 allelic differences, a level previously identified as indicative of potential *E. coli* transmission [48], 105 putative clusters of transmission were identified and comprised isolates from 2 distinct patients or more (Additional file 1: Table S1). Clusters of serial isolates from single patients were removed. Second, to further investigate these putative clusters, an internal reference genome (first isolate temporally) was picked and whole-genome SNP analysis was individually performed for the 105 clusters. Using a 17 SNP cutoff, a threshold previously identified between patient pairs sharing strong epidemiological links [9], 95 of the 105 original clusters were confirmed and were further analyzed in this report. To determine the prevalence of MDR isolates in the clusters, primary MDR cluster isolates (*n*=228) were used (serial isolates from the same patient and same MDR or non-MDR designation were removed) (Table 1).

## Results

### Isolate collection and population structure

Between October 2019 and September 2020, a set of 2075 *E. coli* were collected from all 1776 patients who received care within the National Capitol Region healthcare network (located on the East coast of the USA) (Additional file 1: Table S1). While obtained from 21 facilities, the majority (59%) of the isolates originated from a single, large tertiary care hospital that also served as the central microbiology hub for the remaining 20 facilities. This sampling represents >99% of all *E. coli* cultured from clinical specimens at the central microbiology laboratory during this 1-year period. Isolates were primarily obtained from urine (93%), followed by bloodstream infections (2%), wound infections (2%), and perirectal swabs (1%). A small number of isolates were cultured from fluid (.07%), tissue (.04%), and respiratory (.01%) cultures (Additional file 1: Table S1).

**Fig. 1:**
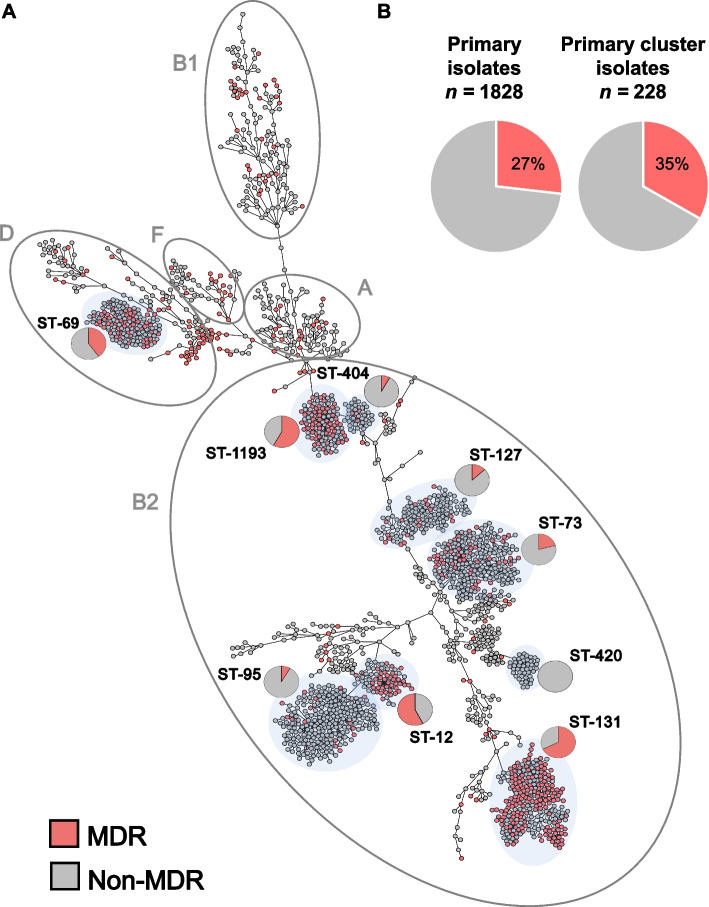
Population structure of a complete collection of *E. coli* clinical isolates for a 1-year period at a US hospital. **A** cgMLST-based minimum spanning tree of 2075 *E. coli* isolates. Isolates belonging to the main phylogenetic groups observed in this study are circled and labeled. The dominant STs are shaded in light gray and the proportion of MDR (red) and non-MDR (gray) isolates within specific STs is indicated by pie charts. **B** Pie charts indicate the prevalence of MDR (red) primary isolates (27%) was similar to the prevalence of MDR isolates in primary isolates predicted to be part of clusters of transmission (35%) (Table 1)

WGS and cgMLST analysis revealed a diverse population that resolved into 5 main *E. coli* phylogenetic groups (Fig. 1A, Additional file 1: Table S1), with B2 and D the most represented (71% and 13%, respectively). Molecular typing by in silico MLST indicated the population was composed of 247 STs with 53% belonging to 6 known, globally prevalent STs. These include the epidemic lineages ST-131 (*n* = 275), ST-73 (*n* = 224), ST-95 (*n* = 215), ST-127 (*n* = 138), and the emerging ST-1193 (*n* = 112) all within phylogroup B2 [49]. Epidemic lineage ST-69 (*n* = 142) was the sole exception, belonging to phylogroup D. Notably, 133 (53%) STs were each found in isolate(s) from single patients and only 52 of 1776 patients carried strains with multiple STs (Additional file 1: Table S1).

### Diversity of antibiotic susceptibility profiles

**Fig. 2:**
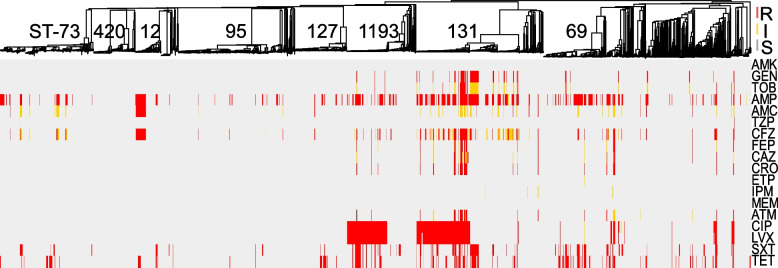
Comprehensive phenotypic antibiotic susceptibility testing of all *E. coli* isolates to 18 antibiotics from 11 classes tested in this study. Breakpoints were interpreted using CLSI guidelines and S (susceptible), I (intermediate), and R (resistant) classifications are labeled for each antibiotic/isolate: red, yellow, and gray, respectively. Interpretations are mapped onto the MST from Fig. 1. AMK amikacin, GEN gentamicin, TOB tobramycin, AMP ampicillin, AMC amoxicillin-clavulanic acid, TZP piperacillin-tazobactam, CFZ cefazolin, FEP cefepime, CAZ ceftazidime, CRO ceftriaxone, ETP ertapenem, IPM imipenem, MEM meropenem, ATM aztreonam, CIP ciprofloxacin, LVX levofloxacin, SXT trimethoprim-sulfamethoxazole, TET tetracycline

Comprehensive AST was performed on all isolates using 18 antibiotics from 11 different classes (Fig. 2, Additional file 2: Table S2). For an accurate determination of the prevalence of resistances in this *E. coli* population, removal of serial isolates (same ST per patient) resulted in a collection of 1828 primary isolates (Table 1). From these, the highest prevalence of non-susceptibility was to ampicillin (41%), followed by tetracycline (23%), trimethoprim/sulfamethoxazole (20%), and fluoroquinolones (15% to ciprofloxacin). In contrast, all isolates were susceptible to amikacin, 5% of *E. coli* were non-susceptible to third- and fourth-generation cephalosporins and <1% (*n* = 14) showed non-susceptibility to a carbapenem. Of the latter, 6 were resistant to imipenem only (MIC = 2), 5 were resistant to ertapenem only (MIC > 0.5 ml/l), 3 were resistant to ertapenem and imipenem or meropenem, and none carried a carbapenemase (Fig. 2, Table 1 and Additional file 2: Table S2).

Distinct lineages of *E. coli* were enriched for phenotypic resistance to various classes of antibiotics: (i) 50% of ST-12 were non-susceptible to amoxicillin/clavulanate (vs. 13% across all isolates, *p* < 0.001 by Fisher exact test), (ii) ST-131 accounted for 59% of isolates non-susceptible to gentamicin and tobramycin (vs. 6% for all, *p* < 0.001), and (iii) ST-131 and ST-1193 alone represented 72% of all isolates with resistance to the fluoroquinolones (Fig. 2, Additional file 2: Table S2).

Overall, 27% of the primary isolates were classified as multidrug resistant (MDR) as defined by Magiorkas et al. (i.e., non-susceptible to at least one agent in ≥3 antibiotic categories) [50] (Fig. 1A and Additional file 2: Table S2), though the prevalence of MDR varied significantly among the distinct, most frequent *E. coli* lineages. For example, while lineages ST-131, ST-1193, and ST-69 were significantly enriched in MDR isolates (64%, 55%, and 43%, respectively, with *p*-values < 0.01 by Fisher exact test), lineages ST-73, ST-127, and ST-95 largely comprised non-MDR isolates (21%, 13%, and 7%, respectively, with *p*-values < 0.03) (Fig. 1A).

### Genomic characterization of ESBL-carrying *E. coli*

**Fig. 3:**
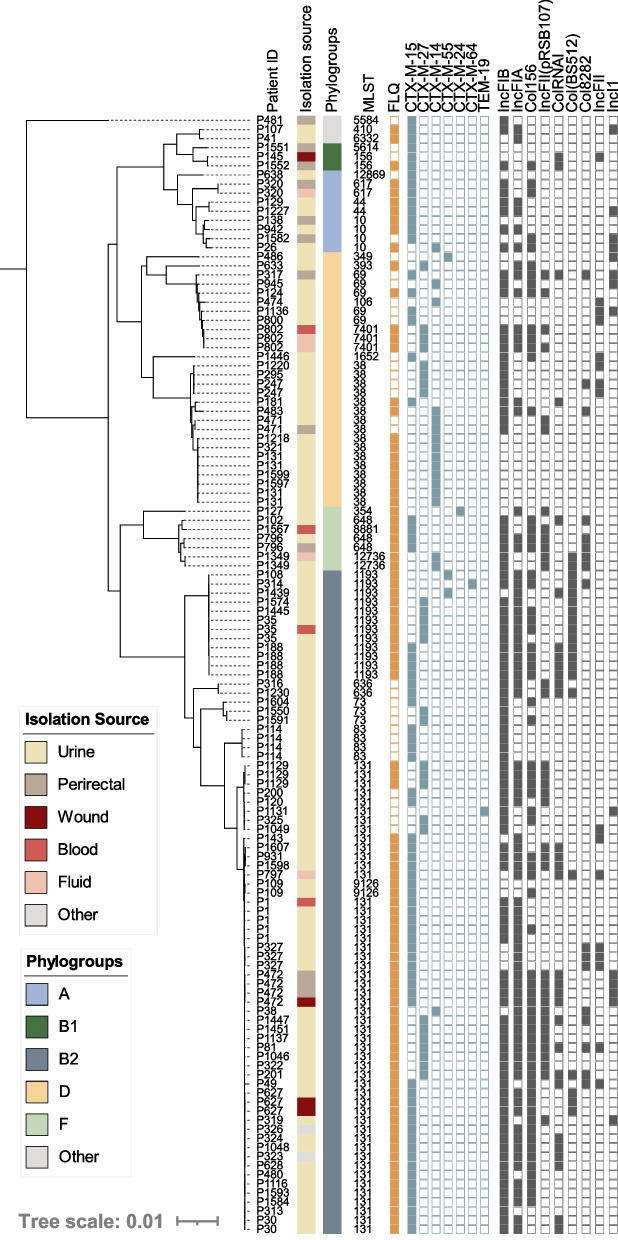
Core genome phylogeny of all ESBL-carrying *E. coli* isolates in our dataset (*n*=123). Patient numbers are listed to identify serial isolates. Isolation source and phylogroups are color coded, indicated by the corresponding legends. Fluoroquinolone (FLQ) (ciprofloxacin and/or levofloxacin) non-susceptibility is indicated by a closed orange square, the presences of unique ESBL alleles are shown in a closed blue square, and plasmid replicon families identified with prevalence ≥10% are indicated by a gray closed square. Two novel ESBL-producing STs were characterized: ST-12869 and ST-12736

During the study period, 123 ESBL-producing *E. coli* were identified from 90 unique patients and all were classified as MDR (Additional file 3: Table S3). Interestingly, 22% were cultured from non-urinary sites, a significant divergence from the overall population (7%, *p*<0.05). Phylogenomic analysis of all ESBL-*E. coli* isolates indicated ESBL producers were diverse and belonged to 26 STs, including prevalent lineages [ST-131 (from 36 patients), ST-1193 (7 patients), ST-69 (5 patients)], less common lineages in our dataset [ST-38 (11 patients), ST-10 (4 patients)], and rarer ESBL-carrying lineages [ST-44 [51], ST-256, and ST-636 [52] each represented by 2 patients each]. As a result, an overrepresentation of ST-131 and ST-1193, which have fluoroquinolone resistance rates of 52% and 100%, respectively, 74% of ESBL-producers were non-susceptible to fluoroquinolones (compared to 17% overall, *p* < 0.01) (Fig. 3). The most represented ESBL genes were *bla*_CTX-M-15_ (59%) and *bla*_CTX-M-27_ (22%). Furthermore, *bla*_CTX-M-14_ was carried by 14% of the isolates including eight ST-38 isolates from 5 patients without an identified plasmid replicon. While carriage on a plasmid with an unknown replicon cannot be ruled out, chromosomal carriage of *bla*_CTX-M-14_ has previously been described for strains of this ST collected from Mongolian birds [53]. *bla*_CTX-M-55_ was observed in 3 isolates and *bla*_CTX-M-24_ and *bla*_TEM-19_ were observed once in distinct lineages (ST-354 and ST-131, respectively) (Fig. 3). Notably, the first description of ST-1193 harboring a *bla*_CTX-M-64_ allele was observed in a singular isolate (Fig. 3). Nine plasmid replicon types regularly associated with ESBL carriage [54, 55] were identified with varying prevalence, from ≥10 to 76% (Fig. 3).

### Outbreak detection reveals the role of non-MDR *E. coli* in nosocomial transmission

**Fig. 4:**
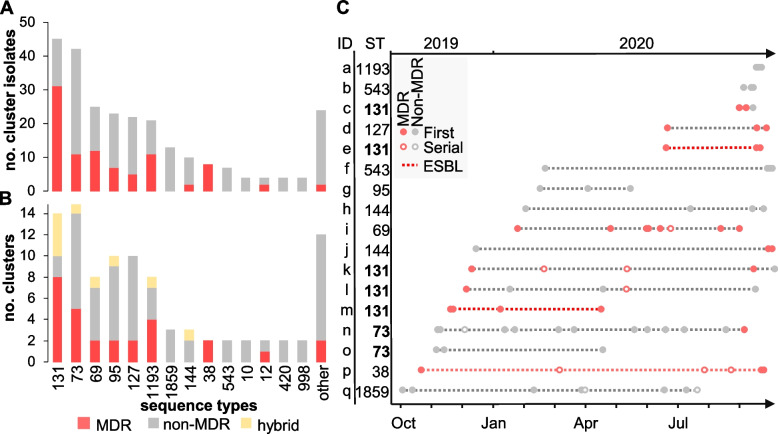
Potential clusters of transmission were defined as groups of isolates from ≥2 patients with ≤17 SNP differences. **A** Stacked histograms showing the number of MDR and non-MDR cluster isolates according to their ST and **B** the number of distinct outbreak clusters per ST. Clusters grouping either MDR or non-MDR isolates are shown in red and gray, respectively. Hybrid clusters (i.e*.*, grouping both MDR and non-MDR isolates) are shown in yellow. STs associated with a single cluster were grouped into others and represent ST-244, ST-394, ST-62, ST-372, ST-404, ST-421, ST-428, ST-538, ST-607, ST-1431, ST-1597, and ST-7887. **C** Analysis of outbreak clusters (identified on the *y*-axis with the corresponding ST) involving ≥3 patients (*n* = 24) and ordered temporally. The legend describes novel patients (filled circles), serial isolates (open circles), MDR isolates (red-filled circles), non-MDR (black-filled circles), and outbreaks consisting of all ESBL-*E. coli* isolates (dashed red line)

Prediction of possible clusters of transmission was performed in two steps: cgMLST followed by SNP analysis (Table 1). This filtering stringently confirmed 95 clusters (from 105 identified by cgMLST) comprising 258 isolates from 227 patients (Table 1). A total of 26 STs were represented and 61% of the clusters (58 out of 95) were caused by a non-MDR clone (Fig. 4A, B and Additional file 1: Table S1). At the isolate level, the proportion of primary MDR (35%) isolates from potential outbreaks clusters was slightly increased compared to primary non-cluster isolates (27%, *p* = 0.007 by Fisher exact test) while the proportion of ESBL producers remained comparable (6%) (Fig. 1B) (Table 1). At the lineage level, the largest number of outbreak clusters involved ST-131 (with 8/14 clusters caused by a MDR clone) and ST-73 (with 9/15 clusters caused by a non-MDR clone) (Fig. 4B).

While the majority of clusters (78 out of 95) were composed of only two patients (an amount of transmission that routine surveillance cannot influence), the remaining outbreaks involved 3 to 12 patients (Fig. 4C). Temporally, these clusters extended up to 11 months, and lineage ST-131 was once again the most represented, with 5 distinct outbreak clones including two (clusters e and m) that were ESBL-producers (Fig. 4C). The largest predicted outbreak involved 12 patients (cluster n) and was caused by a ST-73 clone that was largely non-MDR and cultured primarily from urine (Fig. 4C). The only exception was MDR isolate 836616 which was distinct by only 12 SNPs from non-MDR isolate 822264 from another patient in this cluster and uniquely acquired resistance genes *bla*_*TEM-1*_, *sul2*, *aph(3)-lb*, and *aph(6)-ld* (Additional file 3: Table S3).

### Convergence of resistance and virulence determinants in ST-131 *E. coli*

**Fig. 5:**
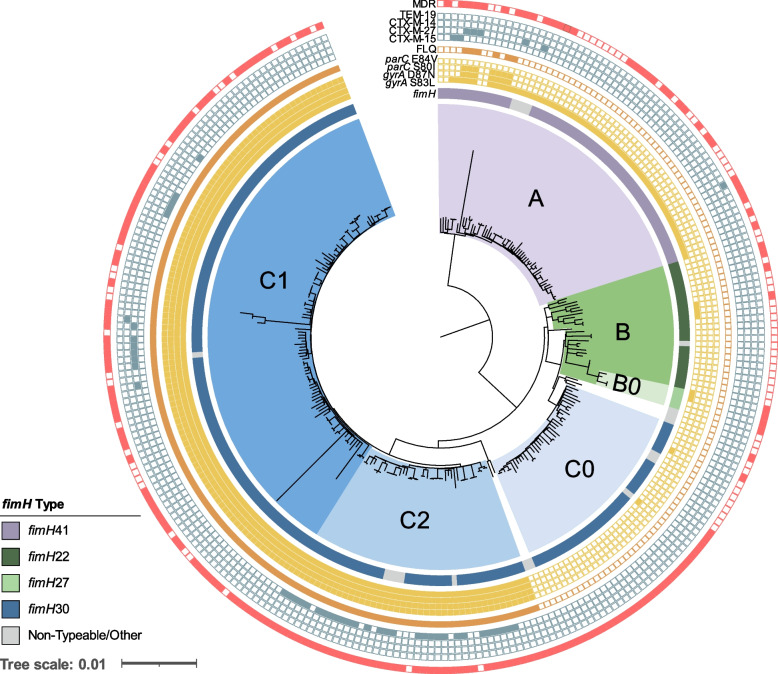
Core genome SNP-based phylogeny of all ST-131 *E. coli* isolates in our dataset (*n*=275). Labels for clades A, B, B0, C0, C2, and C1 are indicated and are colored purple, green, light green, light blue, blue, and dark blue, respectively. Metadata are represented as rings from inner to outer: variations in the *fimH* gene, presence of point mutations in *gyrA* and *parC* (filled yellow square), fluoroquinolone (ciprofloxacin and/or levofloxacin) non-susceptibility (closed orange square), presence of ESBL gene (closed blue square), and multidrug-resistant isolate (red closed square). Non-typeable *fimH* alleles due to truncation or missing gene were grouped with other rare variants identified (Additional file 1: Table S1)

Considering the role played by ST-131 in both outbreak and sporadic infections, a detailed genetic analysis of the resistance and virulence genes found in these US isolates was performed. A maximum-likelihood core SNP-based phylogeny of all *E. coli* ST-131 genomes (*n*=275) in our dataset resulted in the 3 dominant ST-131 clades: clade A (*n* = 59, 21%), clade B (*n* = 29, 11%), and clade C (*n* = 181, 66%) (Fig. 5). Ninety-three percent of clade A isolates carried *fimH*41, 84% of clade B carried *fimH*22, all subclade B0 isolates carried *fimH*27, and 95% of clade C isolates carried the *fimH*30 variant. Of note, 19 isolates had non-typeable *fimH* alleles or a divergent allele designation (Additional file 1: Table S1).

Th predominant clade C isolates were further classified into subclades C0 (*n* = 38), C1 (*n* = 101), and C2 (*n* = 42) (Fig. 5). Unlike clade A, B, and C0 isolates, which were largely (94%) fluoroquinolone susceptible, 100% of clades C1 and C2 isolates carried double *gyrA* and *parC* mutations associated with high-level resistance (Fig. 5) [56]. Furthermore, clade C2 was enriched for ESBL-producing isolates (69% of C2 isolates were ESBL compared to only 9% in other clades) and all carried *bla*_CTX-M-15_. Interestingly, 74% of clade C0 isolates were characterized as MDR (Fig. 5) despite being susceptible to fluoroquinolone and cephalosporin antibiotics. This was largely due to a higher prevalence of resistance to aminoglycosides (68% vs. 30% in all), folate pathway inhibitors (68% vs. 38%), and tetracycline (61% vs. 35%) in comparison to other clades (Additional file 2: Table S2).

In addition to the enrichment of resistance genes, isolates in lineage ST-131 frequently (>80% and *p* < 0.01) carried virulence-associated genes previously identified and associated with ExPEC *E. coli* [57, 58], including the aerobactin locus (*iucC* and *iutA* otherwise found in ~34% of all *E. coli*), a secreted autotransporter toxin (*sat*, 25% in the whole population), and an IrgA-like adhesin (*iha*, 26% in other *E. coli*) (Additional file 4: Table S4).

### Accumulation of virulence genes in non-MDR ST-73 lineage

Together with ST-131, isolates belonging to ST-73 played a prominent role in both sporadic infections and possible cases of nosocomial transmission. However, unlike ST-131, no enrichment of antimicrobial resistance determinants was observed within this lineage, and ST-73 isolates remained largely susceptible to aminoglycosides, cephalosporins, and fluoroquinolones (Fig. 2, Additional file 2: Table S2). In contrast, ST-73 isolates contained significantly (*p* < 0.01) more virulence-associated genes (between 239 and 314) than ST-131 *E. coli* (between 201 and 309) [13] (Additional file 4: Table S4). Specifically, ST-73 isolates were enriched (>70% vs. <25% in the population as a whole) in uropathogenicity-associated virulence factors involved in invasion and colonization (*pic*, *hek*), cell lysis (*hlyA*), and adhesion and penetration (*foc/sfa* and *cnf*) [13, 57, 59] (Additional file 4: Table S4). When compared to the ST-131 population, ST-73 isolates were significantly enriched in a distinct set of virulence genes most likely contributing to the epidemiological success of the lineage (Additional file 4: Table S4).

## Discussion

A significant strength of this study lies in the >99% collection of all *E. coli* isolates from clinical samples over a recent (2019–2020) 12-month period in a network of US military healthcare facilities. Together with comprehensive AST and WGS, this dataset offered a unique opportunity to describe (i) the continued success and emergence of high-risk ExPEC and UPEC lineages, (ii) the regional prevalence of phenotypic resistances (and associated, acquired antibiotic resistance determinants), and (iii) the respective burden of ESBL, MDR, and non-MDR clones in infections of likely nosocomial origin.

Unlike the UK [60, 61], Canada [18], and other regions of the world [62, 63], recent genomic surveillance data on circulating *E. coli* lineages and resistances in the USA is limited. At a global scale, our analysis of this set of US isolates is consistent with previous epidemiological studies demonstrating the predominance (>50% of cases) of ST-69, 73, 95, 127, and 131 pandemic ExPEC lineages [16, 17]. *E. coli* is the world’s leading cause of UTIs, and this is reflected in our collection, where 93% of isolates were from urine samples. The distribution of major lineages observed globally and here also mirrors genomic epidemiology studies of community-acquired (CA)-UTIs across Canada (2012–2015) and from UPEC isolates collected at a Northern California university in 1999–2000 and again in 2016–2017 [18, 19]. However, in contrast to these studies, our collection revealed the emergence of ST-1193 fluoroquinolone-resistant *E. coli* as one of the most prevalent lineages currently circulating in this region of the USA.

Over the past 20 years in the USA, fluoroquinolones have replaced trimethoprim-sulfamethoxazole as the treatment of choice for uncomplicated UTIs [64]. In our collection, fluoroquinolone non-susceptible isolates largely belonged to only two lineages, ST-131 and ST-1193 (72% between both lineages). For ST-131, numerous studies have described the rapid, global emergence and dominance of subclones with acquired fluoroquinolone resistance mutations (subclade C1/H30-R) and a high prevalence of ESBL enzymes (C2/H30-Rx) [16, 17]. In this study, we show that the prevalence of C1 and C2 in the USA (both as an aggregate [52% of ST-131 isolates] and separately with 37% and 15%, respectively) is comparable to estimates from a recent report of a longitudinal collection of *E. coli* (albeit of bloodstream isolates) from the last two decades in Norway [62]. Interestingly, these are also similar to earlier US estimates [collection of 261 isolates from 2010 to 2012 [16]] suggesting the ST-131 population structure has remained relatively stable over the last decade and the overall prevalence of this lineage appears to have plateaued. In contrast, lineage ST-1193, which is the only other known clone driving the spread of fluoroquinolone-resistant *E. coli* globally [65–68], appears to be surging. For example, though the first worldwide cases of ST-1193 only appeared in 2011 [68, 69], a recent US-based multicenter surveillance study of 6349 clinical *E. coli* showed that the fraction of fluoroquinolone-resistant ST-1193 increased from 18 to 25% between 2016 and 2017 [67]. In our study of isolates from 2019 to 2020, the fraction was 31%, suggesting the rapid rise of ST-1193 is still ongoing. At the molecular level, all ST-1193 in this collection carried three characteristic, non-synonymous mutations resulting in high-level fluoroquinolone resistance; ParC (S80I) and GyrA (D87N and S83L) acquired via homologous recombination from a single transfer event at the origins of that lineage [70]. In addition, a fourth substitution in ParE (L416F) previously described in ST-1193 lineage was found in all isolates [65].

In our collection, 5% of primary isolates were ESBL-producers and, of particular concern for treatment regiments, a subset of 74% were co-resistant to the fluoroquinolones. These rates were comparable to the prevalence of resistances observed in a large (>1.5 million isolates), multicenter study of community-onset UTI in the USA over the last decade (6.4% ESBL-producers and 21% fluoroquinolone non-susceptible) [71]. In contrast, another nationwide US study focused on HAIs during a similar timeframe reported substantially higher rates of resistance to fluoroquinolone (35%) and extended-spectrum cephalosporins (17%) [72]. Globally, the rate of ESBL*-E. coli* varies considerably from >40% in regions such as South America, Southeast Asia, India, and China to ~5 to 20% in Europe, Australia, Canada, and the USA [73]. Furthermore, prevalent lineages carrying ESBLs also vary globally (i.e., ST-648 and ST-410 are underrepresented in our study yet are the most prevalent lineages circulating in intensive care units in Vietnam [74]). Importantly, ST-1193 was the third most frequent source of ESBL-producers in our collection, with 8% (*n* = 7) carrying one of the variously represented alleles (*bla*_CTX-M-15_, *bla*_CTX-M-27_, *bla*_CTX-M-55_, and first report of *bla*_CTX-M-64_ carriage), suggesting multiple introductions. In contrast, ESBL-producers composed 69% of isolates within subclade C2 of ST-131 lineage and all carried the same *bla*_CTX-M-15_, most likely harbored on an IncF-type plasmid as previously characterized [75]. Finally, while other countries including France [76], Japan [77], and Germany [78] have seen an increase in the recently defined subclade C1-M27 ST-131 [77] clinical isolates carrying *bla*_CTX-M-27_, we see a low prevalence of subclade C1 *bla*_CTX-M-27_ carrying isolates in this study.

While surveillance and infection control efforts are often and understandably (i.e., increased morbidity, mortality, and financial costs) focused on ESBL and MDR *E.coli* lineages, the global burden of colonization/infection with non-MDR strains (e.g., global lineages ST-73, ST-95, and ST-127) remains invariably higher [5, 62]. In fact, in this cohort of 1776 patients, 3 out of 4 individuals were diagnosed with a non-MDR isolate (representing a diversity of *E. coli* lineages, most of which have yet to be explored). When focusing on patients where in-depth comparative genomics suggested nosocomial origin was likely (*n* = 227), a slight increase in the fraction of MDR cases is observed, but the majority (2 out of 3 patients) were still due to a non-MDR clone. In fact, ExPEC pandemic lineage ST-73 was largely comprised of non-MDR isolates and was both one of the most frequent sources of potential clusters of transmission and responsible for the largest predicted outbreak involving 12 patients. While the possibility of transmission happening outside the hospital (e.g., shared long-term facilities or elderly care home) cannot be excluded, these findings highlight the importance of surveilling *E. coli* isolates with diverse susceptibility profiles as investigations that focus on MDR only are likely to underestimate ongoing outbreaks in the patient population.

To our knowledge, just a single study has performed similar genome-based detection (albeit using a different methodology) of nosocomial transmission on a complete collection of clinical *E. coli* isolates [9]. That study examined stool samples from 97 inpatients over a 6-month period at a single UK hospital. Similar to our findings, the two largest clusters identified spanned the entirety of the study period and were caused by the nosocomial spread of non-MDR isolates. Interestingly, these clones were identified as ST-7095 (7 patients, 29 isolates) and ST-635 (4 patients, 18 isolates) [9], two lineages comprised within phylogroup A that were not detected in our sampling. Whether the epidemic success of these non-MDR lineages simply stems from their overall abundance or could result from the acquisition of virulence/colonization factors (as observed here for ST-73) remains to be fully characterized.

## Conclusions

By capturing all clinical isolates for a full year, this study provides a rare and contemporary survey of the genomic landscape of MDR and non-MDR *E. coli* lineages in a large healthcare network in the Northeast US. While pandemic ST-131 and expanding ST-1193 lineages (both characterized by high rates of co-resistance to fluoroquinolones and extended-spectrum cephalosporins) warrant particular surveillance, our findings also indicate that non-MDR lineages play a significant role in nosocomial transmission. With WGS developing as a near-routine technology in infection control, such improved understanding of the epidemiology of hospital-acquired pathogens is critical for maximum effectiveness at reducing infections and healthcare-associated costs.

## Supplementary Information

**Additional file 1: Table S1.** Basic Isolate Metadata.

**Additional file 2: Table S2.** Antibiotic Susceptibility Testing.

**Additional file 3: Table S3.** AMR Genetic Characteristics.

**Additional file 4: Table S4.** Virulence Associated Genes.

## Data Availability

Both genomic assemblies and raw sequencing data of all isolates analyzed in this study are publicly available in the NCBI database under the BioProject number PRJNA809394 [79].

## Abbreviations

ESBL: Extended-spectrum β-lactamase
MDR: Multidrug resistant
MLST: Multilocus sequence typing
ST: Sequence type
cgMLST: Core genome multilocus sequence typing
NCBI: National Center for Biotechnology Information
